# Variable ventilation with two PEEP Levels (BiPEEP) in patients with acute respiratory distress syndrome: a pilot study^☆^

**DOI:** 10.1016/j.bjane.2025.844673

**Published:** 2025-08-26

**Authors:** Paula C. Fontela, Luiz Alberto Forgiarini Junior, Cristiano Feijó Andrade, Guillermo Bugedo, Gilberto Friedman

**Affiliations:** a Universidade Federal do Rio Grande do Sul (UFRGS), Faculdade de Medicina, Programa de Pós-Graduação em Ciências Pneumológicas, Porto Alegre, RS, Brazil; b Universidade Católica de Pelotas (UCPel), Pelotas, RS, Brasil; c Hospital de Clinicas de Porto Alegre, Laboratório de Vias Aéreas (VAP), Porto Alegre, RS, Brasil; d Pontificia Universidad Católica de Chile, School of Medicine, Chile

Patients with Acute Respiratory Distress Syndrome (ARDS) commonly require Mechanical Ventilation (MV) to restore or maintain adequate oxygenation when critically ill. Previous studies suggest that Variable Ventilation (VV) is able to induce pulmonary recruitment,^1^ and especially to prevent alveolar derecruitment.^2^^,^^3^ This mechanism is of paramount importance in ARDS, when the major challenge is not just recruiting the injured lung, but maintaining recruitment when protective mechanical ventilation is advocated. No previous study has used PEEP as a variability variable. Based on experimental studies,^2^, ^3^, ^4^ our hypothesis is that in the short term, VV with two PEEP levels (BiPEEP) would result in comparable gas exchange, better respiratory mechanics without changing hemodynamics.

We performed a crossover randomized clinical trial with return. This study was reviewed and approved by the Research Ethics Committee of the Santa Casa Hospital Complex of Porto Alegre (registry 928.,427) and is registered in the Brazilian Registry of Clinical Trials (RBR-5bb65v).

The study population included 8 patients admitted to the intensive care unit who met the following inclusion criteria: age > 18 years, mechanical ventilation > 24 hours, diffuse infiltrate on chest X-Ray, arterial partial Pressure of Oxygen/Fraction of Inspired Oxygen (PaO_2_/FiO_2_) ratio 100‒300 mmHg. Patients were excluded if they presented lung emphysema, pneumothorax or lung barotrauma of any kind, and chest drain. Informed consent was obtained from family members or caregivers as soon as they were eligible for the study. Clinical data, current therapy and diagnosis were reviewed and obtained through the ICU electronic data system.

During the three-hour study period, all patients were ventilated with Conventional Ventilation (CV) and variable ventilation with two levels of PEEP (BiPEEP) for one hour each, alternating them randomly. Randomization was performed on the website www.randomization.com, with a 1:1 allocation frequency using blocks of 4 patients to determine the sequence of MV modes (BiPEEP - CV - BiPEEP or CV - BiPEEP - CV). The assessor was not blinded to the randomization of the ventilation mode. Data analysis was performed by a blinded assessor. This methodology was used to assess whether the effects of ventilatory modes return to their baseline patterns.

All patients were monitored with continuous electrocardiogram, pulse oximetry, and invasive blood pressure. Once the intensivist in charge and the investigators considered it safe, each patient was transferred to the study ventilator and a 10-minute period was given prior to baseline measurements. After baseline measurements, the MV sequence was randomized and patients were ventilated for three consecutive hours, with a 10-minute wash-out period, alternating CV with BiPEEP, one hour in each ventilatory mode. The study was discontinued if any of the following criteria were present: increased Heart Rate (HR) > 20% compared to baseline, or < 50 bpm, or > 130 bpm; increase in MAP > 20% from baseline, or < 60 mmHg, or > 110 mmHg. After the study protocol was completed, patients were returned to the previous mechanical ventilator. Ventilatory mechanics and arterial blood gas were obtained at the beginning and end of each of the three ventilation periods. The Intermed 7 Plus® ventilator (CareFusion, São Paulo, Brazil) was used for both CV and BiPEEP modes. Both MV modes were performed in Pressure-Controlled Ventilation (PCV). The mechanical ventilation was set to a VT of 6 mL.kg^−1^ of predicted weight and a peak airway pressure ≤ 35 cm H_2_O, respiratory rate of 20 breaths/min, inspiratory Time (Ti) of 1.0 second and 100% Inspired Oxygen Fraction (FiO_2_). To perform ventilation with BiPEEP, the mechanical ventilator has an adjustment that allows the automatic elevation of PEEP: the baseline PEEP used in BiPEEP was 5 cm H_2_O and it was automatically increased to 10 cm H_2_O every four ventilatory cycles. PEEP switching was fully automated.

Data are presented as mean and standard error. The comparison between VC and BiPEEP was performed using ANOVA ‒ Latin Square 2×3 to counteract the effects of patient variability over time, each ventilation in each patient was tested once each time. Thus, the baseline of the two types of ventilation (treatment) was analyzed in three periods of one hour (sequence). Therefore, baseline, treatment and sequence analyses are presented, in which the difference in the variables was verified according to the randomization of the treatment sequence. A Latin square is a design used in experiments in which each subject is measured in each treatment and changes in conditions need to be controlled. It is a design in which each treatment is assigned to each time period and to each subject an equal number of times. All tests were performed using the Statistical Package for the Social Sciences (version 19.0). Statistical significance was accepted with a p < 0.05. The data showed normal distribution.

All patients (4 men/4 women) were between 34 and 78 years of age, and PaO_2_/FiO_2_ on inclusion ranged from 119 to 204. All patients completed all phases and even if any data points were excluded, and all patients tolerated the intervention without protocol violations or adverse events.

Gas exchange and acid-base parameters did not differ between CV and BiPEEP. HR significantly decreased during BiPEEP. Initiating MV with CV significantly reduced MAP compared to BiPEEP. However, this change was not clinically significant and there was no need to interrupt the protocol. Among the gas exchange, hemodynamic and acid-base balance variables, only SpO_2_ showed carryover (p = 0.025).

Pressure level (∆ pressure) and Peak airway Pressure (PIP), as well as expiratory resistance, VT and minute ventilation did not differ between CV and BiPEEP. Static pulmonary compliance was significantly higher with BiPEEP, while Pplat and Driving Pressure (DP) increased compared to CV (Fig. 1A‒G). Starting the MV sequence with BiPEEP significantly increased minute ventilation, Pplat, PIP, and DP compared to CV, possibly by the difference in PEEP values between ventilatory modes (Table 1). Among the respiratory mechanic parameters, minute ventilation showed carryover (p = 0.020).

**Figure 1:**
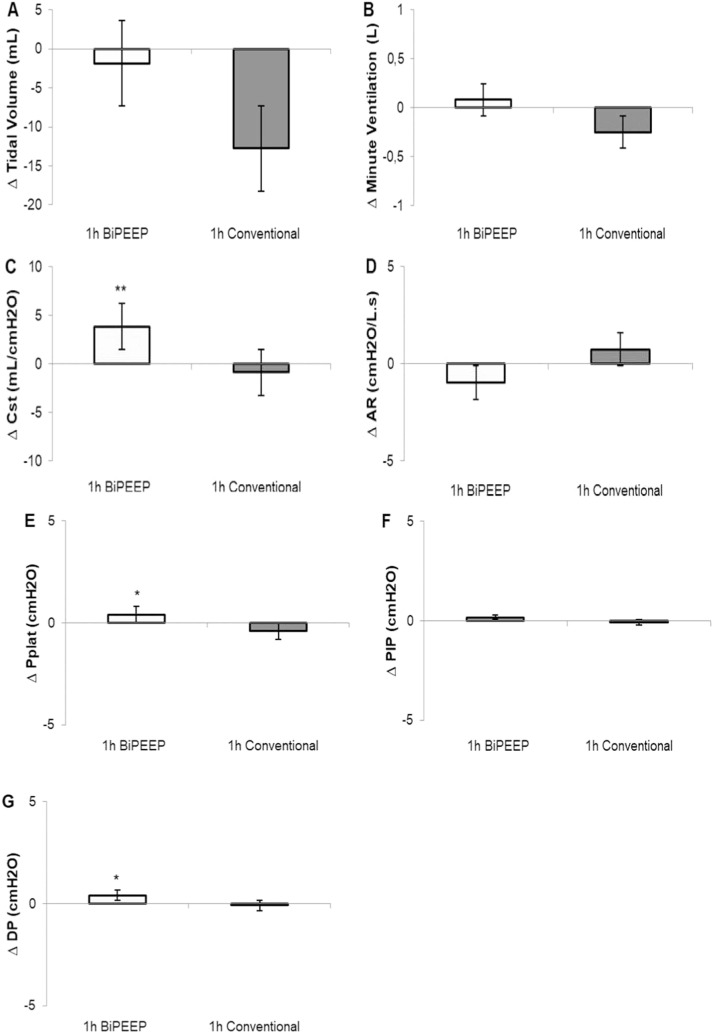
Respiratory mechanics. Respiratory mechanics after one hour of conventional and variable ventilation with BiPEEP. (A) Tidal Volume (VT); (B) Minute Ventilation (VMin.); (C) Static Compliance (Cst); (D) Airway Resistance (AR); (E) Plateau Pressure (Pplat); (F) Peak Pressure (PIP); (G) Driving Pressure (DP). Data are presented as baseline delta (∆) difference between initial and final measurements. Values are showed as mean and standard error (* p < 0.05; ** p < 0.001).

**Table 1:** Respiratory mechanics.

	Basal		Sequence		
	BiPEEP	Conventional	BiPEEP - Conventional - BiPEEP	Conventional - BiPEEP - Conventional	p
**VT (mL)**	382.2±37.8	361.5±37.3	−7.50 (4.30)	−7.08 (3.93)	0.943
**VMin (L)**	8.2±1.8	8.4±1.8	0.13 (0.12)	−0.30 (0.30)	0.001^a^
**Cst (mL.cm^−1^ H_2_O)**	29.4±6.5	28.5±8.2	0.88 (1.2)	2.15 (1.32)	0.479
**Rva (cm H_2_O/L.s)**	26.0±7.9	25.4±8.3	−0.37 (0.43)	0.15 (0.39)	0.364
**Pplat (cm H_2_O)**	18.1±3.6	18.2±4.0	0.41 (0.29)	−0.41 (0.27)	0.039^a^
**PIP (cm H_2_O)**	19.7±3.3	20.2±4.2	0.33 (0.20)	−0.25 (0.21)	0.050^a^
**DP (cm H_2_O)**	13.1±3.6	13.2±4.0	0.41 (0.29)	−0.41 (0.27)	0.039^a^

Sequence data is expressed as delta: the difference between the initial and final measurement.

Baseline Conventional Ventilation ‒ Pressure Controlled Ventilation (PCV) with PEEP 5 cm H_2_O, tidal volume of 6 mL.kg^−1^ of predicted weight and a peak airway pressure ≤ 35 cm H_2_O, respiratory rate of 20 breaths/min, inspiratory time (Ti) of 1.0 second and 100% inspired oxygen fraction (FiO_2_).

Baseline BiPEEP - Pressure Controlled Ventilation (PCV) with PEEP 5 cm H_2_O and every four ventilatory cycles it was automatically increased to 10 cm H_2_O, tidal volume of 6 mL.kg^−1^ of predicted weight and a peak airway pressure ≤ 35 cm H_2_O, respiratory rate of 20 breaths/min, inspiratory Time (Ti) of 1.0 second and 100% inspired oxygen fraction (FiO_2_).

VT, Tidal Volume; VMin, Minute Ventilation; Cst, Static Compliance; Rva, Airway Resistance; Pplat, Plateau Pressure; PIP, Peak Pressure; DP, Driving Pressure. Values are presented as mean and standard deviation.

The comparison between conventional ventilation and BiPEEP was performed using ANOVA of repeated measures (Latin Square 2×3).

a p-value based on the sequence.

This is the first pilot study with a clinical trial design with BiPEEP, to our knowledge. The main finding of the present study was that VV with BiPEEP appears to be safe and viable in patients with mild to moderate ARDS.

The use of VV in experimental ARDS models has shown a consistent improvement in arterial oxygenation, as well as respiratory mechanics.^2^ In this study, gas exchange did not differ significantly between CV and BiPEEP. Our findings are similar to the study using variable support Pressure Ventilation (PSV) in 13 patients with mild to moderate acute hypoxemic respiratory failure,^4^ in which variable PSV was associated with better patient-ventilator synchrony and comparable levels of gas exchange. One possible explanation for the absence of significant gas exchange improvement may have been the relatively short time period in which BiPEEP was employed and thus improvement in lung compliance was not accompanied by a change in gas exchange. In fact, most studies that had positive results on arterial oxygenation applied VV over a period of 3‒6 hours.^2^^,^^5^, ^6^, ^7^

A preclinical study of VV with BiPEEP showed that PEEP variability did not cause new pulmonary and inflammatory structural changes.^8^ In the present study, BiPEEP triggered a significant improvement in static lung compliance, while increasing Pplat and DP. The increase in Pplat secondary to PEEP elevation during VV, although statistically significant, remained below the safety limit for protective ventilation in ARDS_._ In addition, we found no significant difference in VT during ventilation with CV and BiPEEP. The increase in Pplat and DP above safe values are related to increased lung injury caused by mechanical ventilation, and DP is also related to increased risk of mortality.^5^

Interestingly, one group of investigators showed that the type of variability, natural (recorded from subjects) or random (randomly generated by a computer), seems not to play a major role in the effects of VV.^9^

They concluded that the percentage, but not the type of respiratory variability is crucial to VV success. In the present study, PEEP was varied every four respiratory cycles, yielding a variability of approximately 25%. The variability employed in this study is closely related to the physiological variability of the respiratory system.^10^ A possible limitation of the study is related to the ventilation time at each mode (1-hour) that may not have been sufficient to capture significant changes in gas exchange. Furthermore, it was not possible to evaluate outcomes such as recruitment or long-term oxygenation, lack of imaging (e.g., lung ultrasound or computed tomography) or biomarkers to assess derecruitment. No correction for multiple comparisons was performed, which may be considered a limitation of the study. The small sample size limits the generalizability of the results as well as the assessment of feasibility and safety. No power calculations were performed due to the pilot nature of the study. Therefore, larger studies with longer observation periods are needed to validate the present results.

Compared to CV, VV with BiPEEP in a clinical setting, improved static pulmonary compliance with comparable levels of gas exchange. In the short term, BiPEEP appears to be safe and feasible in patients with mild to moderate ARDS.

## Conflicts of interest

The authors declare no conflicts of interest.
